# Mouse models and strain-dependency of Chédiak-Higashi syndrome-associated neurologic dysfunction

**DOI:** 10.1038/s41598-019-42159-0

**Published:** 2019-05-01

**Authors:** Adam Hedberg-Buenz, Laura M. Dutca, Demelza R. Larson, Kacie J. Meyer, Dana A. Soukup, Carly J. van der Heide, Hannah E. Mercer, Kai Wang, Michael G. Anderson

**Affiliations:** 1 VA Center for the Prevention and Treatment of Visual Loss, Iowa City VA Health Care System, Iowa City, IA 52246 USA; 2 Department of Molecular Physiology and Biophysics, The University of Iowa, Iowa City, IA 52242 USA; 3 Department of Biostatistics, The University of Iowa, Iowa City, IA 52242 USA; 4 Department of Ophthalmology and Visual Sciences, The University of Iowa, Iowa City, IA 52242 USA; 5 Present Address: Biology Department, College of St. Benedict & St. John’s University, Collegeville, Minnesota 56321 USA

**Keywords:** Cerebellum, Neurodegenerative diseases

## Abstract

Chédiak-Higashi syndrome (CHS) is a lethal disorder caused by mutations in the *LYST* gene that involves progressive neurologic dysfunction. *Lyst*-mutant mice exhibit neurologic phenotypes that are sensitive to genetic background. On the DBA/2J-, but not on the C57BL/6J-background, *Lyst*-mutant mice exhibit overt tremor phenotypes associated with loss of cerebellar Purkinje cells. Here, we tested whether assays for ataxia could measure this observed strain-dependency, and if so, establish parameters for empowering phenotype- and candidate-driven approaches to identify genetic modifier(s). A composite phenotypic scoring system distinguished phenotypes in *Lyst*-mutants and uncovered a previously unrecognized background difference between wild-type C57BL/6J and DBA/2J mice. Accelerating rotarod performance also distinguished phenotypes in *Lyst*-mutants, but at more advanced ages. These results establish that genetic background, *Lyst* genotype, and age significantly influence the severity of CHS-associated neurologic deficits. Purkinje cell quantifications likewise distinguished phenotypes of *Lyst*-mutant mice, as well as background differences between wild-type C57BL/6J and DBA/2J mice. To aid identification of potential genetic modifier genes causing these effects, we searched public datasets for cerebellar-expressed genes that are differentially expressed and/or contain potentially detrimental genetic variants. From these approaches, *Nos1*, *Prdx*2, *Cbln3*, *Gnb1*, *Pttg1* were confirmed to be differentially expressed and leading candidates.

## Introduction

Chédiak-Higashi syndrome (CHS) is a lethal disorder involving recurrent infections, coagulation defects, hypopigmentation, and progressive neurologic dysfunction^1–5^. CHS is caused by recessive mutations in the *LYST* gene^6–8^. Most cases of CHS are recognized in infants based on partial albinism and recurrent pyrogenic infections^9,10^ and are treated with bone-marrow transplantation^11–13^. Other cases are later onset and do not require bone-marrow transplantation^14–17^. Rarely, CHS initially presents as adult Parkinsonian syndrome, dystonia, or dementia^18–22^. Regardless of when CHS is diagnosed, how it is treated, or its initial severity, all patients reported in the literature to date ultimately develop progressive neurologic deficits, including difficulty walking, loss of balance, and tremor^16,20,23^. For most patients these deficits become debilitating in their early 20’s^23^, independent of whether or not they have received bone-marrow transplantation. Patients with neurologic involvement consistently have cerebellar volume loss^16,23,24^. Unfortunately, there are no existing treatments for the neurologic component of CHS.

One means of discovering potential treatments for the neurodegenerative component of CHS is through mouse genetics. As in humans, mice with mutations in the *Lyst* gene can also develop progressive neurologic deficits^25,26^. In mice, the severity of the neurodegenerative phenotype is highly sensitive to genetic background. Comparing extensively aged (17–20 months in age) C57BL/6J (B6) mice homozygous for the *Lyst*^*bg-J*^ mutation (B6-*Lyst*) to a congenic strain of DBA/2J (D2) mice homozygous for the same mutation (D2.*Lyst*), we have previously observed that mice with a D2 background develop an overt tremor associated with Purkinje cell degeneration, whereas mice with a B6 background remain relatively normal^26^. The factors responsible for this difference remain unknown, but if identified, could shed light on the causes and potential treatments for CHS-associated neurologic disease. Among the challenges prohibitive to identification of the modifier(s), two are particularly noteworthy. First, the neurologic deficits occurring on the D2 background have thus far only been described in qualitative terms with an imprecisely known age of onset^26^, thus hindering phenotype-driven genetic approaches. Second, few genes differentially influencing the cerebellum of D2 versus B6 mice are currently known, thus hindering candidate-driven molecular approaches.

Here, we apply behavioral assays for cerebellar disease combined with quantitative analysis of cellularity in the Purkinje layer to the study of B6-*Lyst* and D2.*Lyst* mice and test their ability to distinguish strain-dependent responses. As an assay with ordinal output, we first apply a composite phenotypic scoring system for mouse models of cerebellar ataxia, hereafter referred to as the (CPSS), developed by Guyenet *et al*.^27^ and show that it can effectively distinguish strain-dependent phenotypes in *Lyst*-mutant mice at all ages, as well as previously unrecognized background differences between wild-type B6 and D2 mice. Next, as an assay with quantitative output, we test accelerating rotarod performance and show that it can distinguish strain-dependent phenotypes in mice 14–16 months of age. Our results from the quantitative analyses of cells in the Purkinje layer of cerebellum were consistent with those of the CPSS and accelerating rotarod assays—that is cellularity is decreased in D2.*Lyst* compared to B6-*Lyst* and in D2 compared to B6 mice. Finally, as a resource to help identify candidate modifier genes potentially responsible for these differences in cerebellar disease, we use informatics and public repositories to identify genes normally expressed in the cerebellum containing potentially detrimental genetic variants between D2 and B6 mice. Using quantitative reverse transcription PCR (RT-qPCR) to independently assess RNA expression levels, five genes are identified as leading candidates.

## Results

### Strain-dependent phenotypes assessed with an ordinal CPSS for cerebellar ataxia

**Figure 1:**
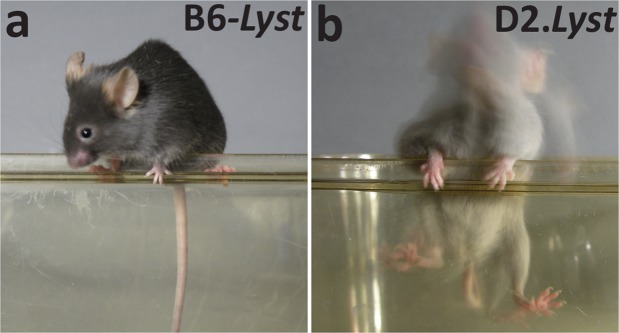
*Lyst* mutation causes strain-dependent neurologic deficits. Long-exposure photographs of mice balancing themselves along the narrow edge of a holding pen illustrating the tremor and ataxic phenotypes of *Lyst*-mutant mice. (**a**) B6-*Lyst* mice do not tremor and are able to maintain balance, resulting in only mild blurring of long-exposure images. (**b**) D2.*Lyst* mice have overt tremor and are unable to maintain balance, resulting in severe blurring of the images. Mice were 19–20 months of age.

**Figure 2:**
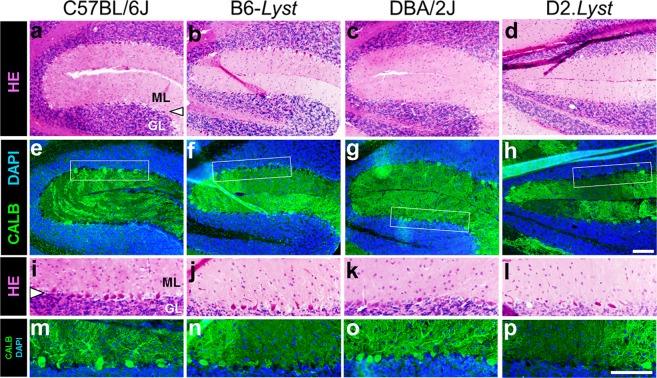
*Lyst* mutation causes strain-dependent loss of cerebellar Purkinje cells. Histological images taken from anatomically-matched positions of cerebellum, stained with H&E- and immunofluorescently-labeled, from aged *Lyst*-mutant and wild-type control mice on the C57BL/6J and DBA/2J inbred backgrounds. All images were taken from serial midsagittal sections of mouse cerebella at low (*top two rows*) and higher magnification (*bottom two rows, with locations denoted by inset white boxes*). Images show qualitative differences in cell density in the cerebellum, where Purkinje cells form a mono-layer (*arrowhead*) between the molecular (*ML*) and granular layers (*GL*). (**a**–**d**) In H&E-stained samples, there are fewer Purkinje cells in *Lyst*-mutant mice than in their strain-matched controls. In normal tissue, Purkinje cells have relatively few and short gaps between adjacent cells. (**e**–**h**) Immunofluorescence-labeled images treated with an antibody labeling the somata and dendrites of Purkinje cells (anti-CALBINDIN, *green*) and a nuclear stain (DAPI, *blue*), again showing a *Lyst*-dependent loss of Purkinje cell somata and dendritic branching, which is particularly notable in D2.*Lyst* mice. **(i**–**p)** In higher magnification views of the same samples, the *Lyst*-dependent cell loss is again evident. The density of dendritic branching in the *ML* is correlative to the number of Purkinje cells. Cell loss is particularly notable in the comparison of DBA/2J and D2.*Lyst* mice (compare panels k versus l; o versus p). All images were anatomically-matched from the inferior cerebella (fissure between lobules IIV and IX) of mice age-matched at 19–20 months of age. Note that in some cases, a lack of staining along the Purkinje layer represents a histological artifact, in which the somata of some Purkinje cells have been pulled away from the section leaving black holes (dendritic labeling without soma labeling), rather than a biological loss of these cells (lack of both dendritic and soma labeling). Some panels (in **i**–**p**) have been flipped vertically to present them all in the same orientation. Key: molecular layer, *ML*; Purkinje cell layer, *arrowhead*; granule cell layer, *GL*. Scale bars = 100 µm.

**Figure 3:**
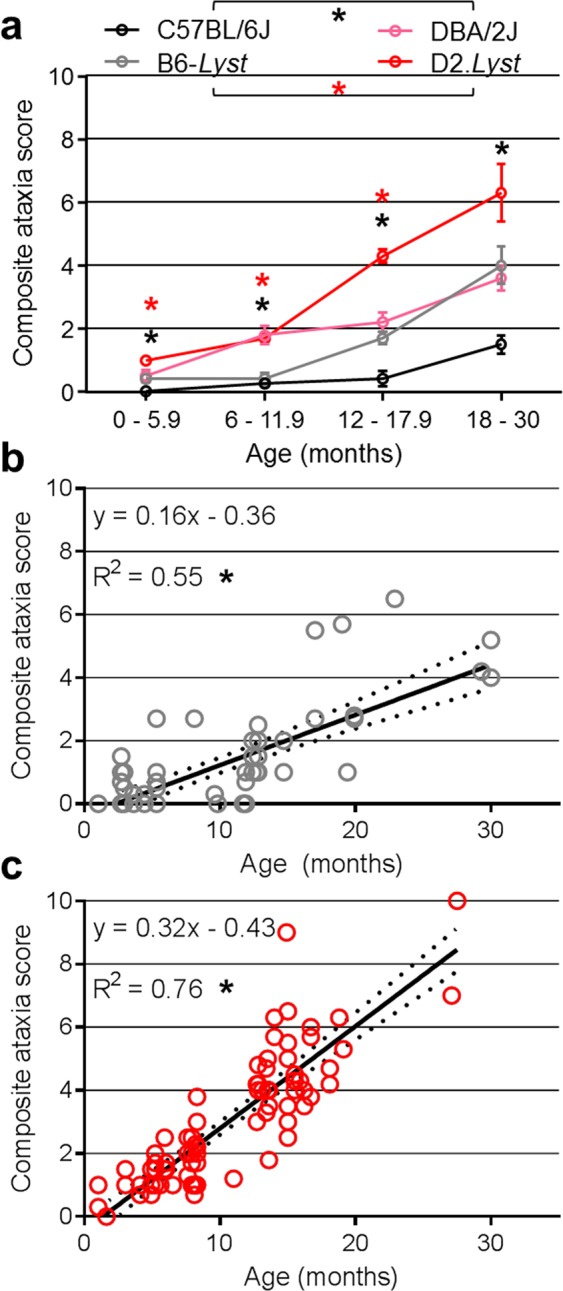
Strain-dependent phenotypes assessed with the CPSS for cerebellar ataxia. (**a**) Graphs of composite scores for each strain at the binned ages indicated; increasing scores are associated with increasing severity of ataxia. At all ages, DBA/2J exhibited significantly higher scores than C57BL/6J mice (*black asterisks, p* ≤ 2.5E^−2^), and D2.*Lyst* had higher scores than B6-*Lyst* mice (*red asterisks, p* ≤ 1.7E^−3^). Data points represent the mean composite score ± SEM for all mice within the indicated group and two-tailed Student’s *t*-test with Bonferroni correction for four tests per comparison. Of the four strains examined, genetic background (DBA/2J, *p* < 1.0E^−4^), *Lyst* genotype (mutant-*Lyst*, *p* < 1.0E^−4^), and age (increasing age, *p* < 1.0E^−4^) significantly affect the severity of scores using linear regression analysis and ANOVA. (**b**) Dot plots of composite scores of individual B6-*Lyst* and (**c**) D2.*Lyst* mice. Data points represent the composite score for one mouse, inset equations describe the linear fit of trend lines and coefficient of determination (*R*^*2*^), dotted lines above and below trend lines represent the 95% confidence interval, and (*) represents *p* < 1.0E^−4^. Numbers of mice within each binned age group (0–5.9, 6–11.9, 12–17.9, 18–30 months) are as follows: D2.*Lyst* (*n* = 30, 27, 39, 6), B6-*Lyst* (*n* = 29, 13, 19, 8), DBA/2J (*n* = 22, 13, 4, 7), and C57BL/6J (*n* = 24, 19, 6, 3).

**Figure 4:**
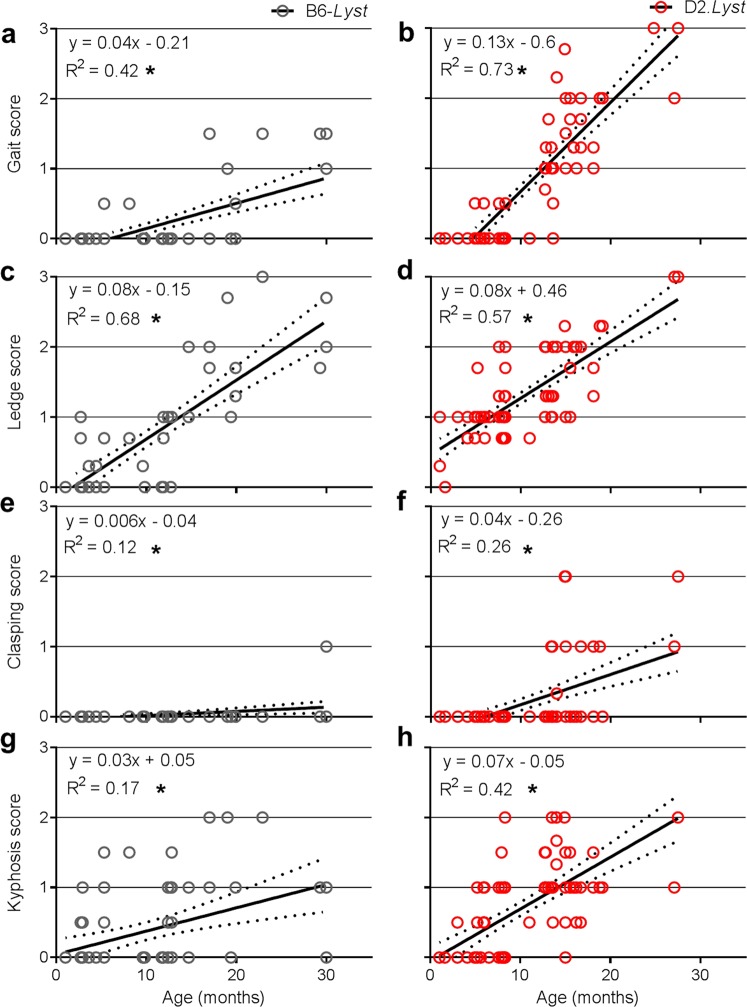
Strain-dependent phenotypes assessed by the four discrete measures within the CPSS for cerebellar ataxia. Graphs of scores, for each of the distinct neurobehavioral tests contributing to the composite score, versus age of: (**a**,**b**) gait, **(c**,**d)** ledge walking, **(e**,**f)** hind limb clasping, and **(g**,**h)** kyphosis for B6-*Lyst* and D2.*Lyst* mice. Note that the trends in score within each measure for D2.*Lyst* mice are similar to, or greater than, those of B6-*Lyst*. Numbers of mice within each binned age group (0–5.9, 6–11.9, 12–17.9, 18–30 months) are as follows: D2.*Lyst* (*n* = 30, 27, 39, 6), B6-*Lyst* (*n* = 29, 13, 19, 8), DBA/2J (*n* = 22, 13, 4, 7), and C57BL/6J (*n* = 24, 19, 6, 3). In all graphs, each data point represents the composite score for one mouse, inset equations describe the linear fit of trend lines and coefficient of determination (*R*^*2*^), dotted lines above and below trend lines represent the 95% confidence interval, and (*) represents *p* < 3.0E^−3^.

We have previously shown that aged D2.*Lyst* mice exhibit a tremor phenotype that is dependent upon genetic background (Fig. 1) and is associated with a loss of cerebellar Purkinje cells (Fig. 2)^26^. To better define the nature of the tremor phenotype exhibited by *Lyst*-mutant mice, especially with respect to age of onset, we compared *Lyst*-mutants from both backgrounds using the CPSS^27^ for mouse models of cerebellar ataxia (Figs 3 and 4). The CPSS sums the results from four individual assays (hind limb clasping, ledge walk, gait, and kyphosis) into one composite score ranging from 0 (unaffected) to 12 (severe). D2.*Lyst* mice had significantly higher composite scores than B6-*Lyst* mice at all ages (0–5.9 months: *p* = *1.7*E^−3^, 6–11.9 months: *p* = *4.4*E^−5^, 12–17.9 months: *p* = *9.4*E^−10^), except at 18–30 months, in which the difference was not statistically significant (*p* = 2*.1*E^−1^, Bonferroni corrected *p*-value using a two-tailed Student’s *t-*test with four comparisons; Fig. 3a). In both genetic backgrounds, *Lyst*-mutants older than 12 months of age showed significantly increased composite scores in comparison to their strain-matched wild-type controls (B6 versus B6-*Lyst*: *p* = *1.1*E^−2^ (for 12–17.9 months), *p* = *6.0*E^−3^ (18–30 months); D2 versus D2.*Lyst*: *p* = *8.0*E^−4^ (12–17.9 months), *p* = *2.6*E^−2^ (18–30 months). Using linear regression analysis, age (*p* < 1.0E^−4^), genotype (*p* < 1.0E^−4^), and genetic background (*p* < 1.0E^−4^) were each found to impart significant influence on composite score. Furthermore, the effect of age significantly interacts with that of genetic background (*p* = 4.8E^−13^) and *Lyst* genotype (*p* = 9.5E^−9^). In examining the metrics of each assay, which contribute to the composite score for each mouse (Fig. 3b,c), both *Lyst*-mutant strains had mean scores >0 for each assay, indicating a multi-factorial basis (Fig. 4a–h). Because strain-dependent differences in *Lyst*-mutant mice were previously only known to be present in 17–20 month old mice, these results indicate that cerebellar deficits occur earlier than previously detected and suggest that the CPSS for cerebellar ataxia could be a useful tool in studying them further.

In order to test *Lyst*-dependency of the age-related ataxia of B6-*Lyst* and D2.*Lyst* mice, the CPSS was also used to compare the B6 and D2 inbred strains with wild-type *Lyst* alleles. D2 mice exhibited significantly greater composite scores than B6 at all ages (0–5.9 months: *p* = *2.0*E^−3^, 6–11.9 months: *p* = *1.9*E^−5^, 12–17.9 months: *p* = *2.5*E^−2^, 18–25.4 months: *p* = *1.8*E^−2^; Bonferroni corrected *p*-value using a two-tailed Student’s *t-*test with four comparisons; Fig. 3a). Trend lines for both *Lyst*-mutant strains qualitatively appeared to parallel those of the respective inbred strains. Thus, the strain-dependent differences observed in *Lyst*-mutants with this assay appear to be a predominantly additive effect, whereby *Lyst* mutation causes a more-or-less uniform perturbation that differs between B6-*Lyst* and D2.*Lyst* mice because of underlying baseline differences between the B6 and D2 strains themselves.

### Strain-dependent phenotypes assessed with a quantitative assessment of motor impairment by accelerating rotarod

**Figure 5:**
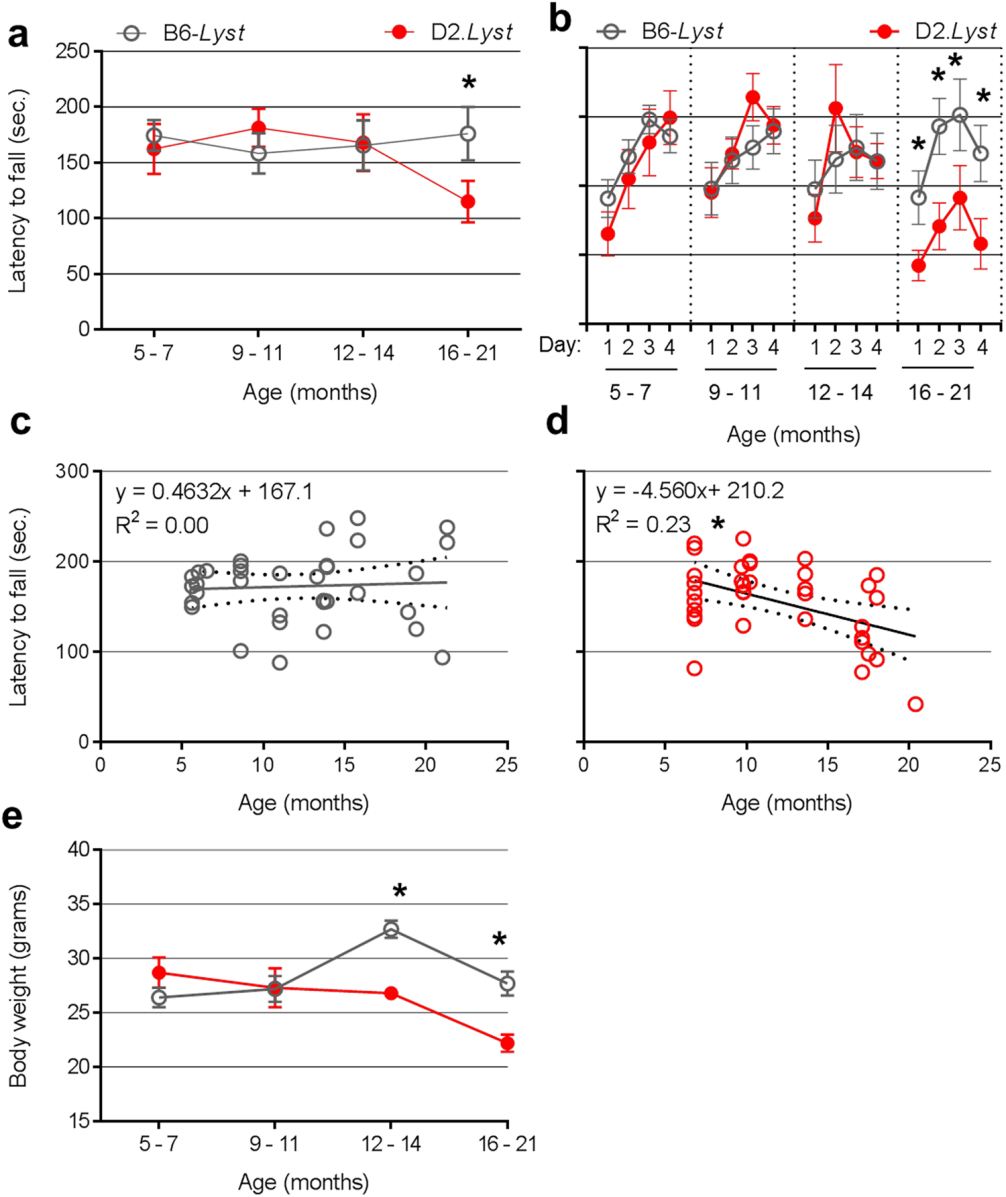
Strain-dependent phenotypes assessed by accelerating rotarod. (**a**) Graph of accelerating rotarod performance expressed as latency to fall plotted against binned age group. Note a trend of decreasing performance in D2.*Lyst* mice, whereas the performance of B6-*Lyst* remains relatively constant with age. Each data point represents mean latency ± SEM, from three consecutive trials per session, on four consecutive days (*n* = 12 trials per mouse) for all individuals of each strain at each age group. (**b**) Graph of rotarod data of mean latency plotted against each of the four consecutive days of testing for each strain at each age showing no significant differences in latency until 16–21 months of age, at which, latency is significantly decreased in D2.*Lyst* compared to B6-*Lyst*. Each data point represents mean latency ± SEM for each day (*n* = 3 trials each day) for all individuals of each strain at each age group. (**c**) Rotarod data (from panel *b*) presented as dot plots with linear regression analysis for all B6-*Lyst* and (**d**) D2.*Lyst* mice. (**e**) Graph of mean body weight ± SEM versus binned age. Numbers (*n* = ) of mice within each binned age group (5–7, 9–11, 12–14, 16–21 months) are as follows: D2.*Lyst* (*n* = 10, 10, 5, 10) and B6-*Lyst* (*n* = 10, 9, 8, 10). For tests of statistical significance, (*) represents: (**a**,**b**,**e**) a Bonferroni-corrected *p* < 0.05 using a two-tailed Student’s *t*-test with 4 tests (one for each matched age group) and (**c**,**d**) linear regression with a one-way ANOVA.

Previous studies of motor coordination with mice have shown that rotarod performance is highly influenced by genetic background^28,29^ and age^30–32^. Studies have shown that young (≤4 months) D2 mice perform similarly to^33,34^, or worse than^28,35,36^ B6 mice with a decreased capacity for motor learning^37^. To our knowledge, no study has assessed the influence that *Lyst*-mutation imparts on accelerating rotarod performance. To test the ability of the accelerating rotarod assay to differentiate strain-dependent phenotypes, *Lyst*-mutant mice were compared using an accelerating rotarod assay (Fig. 5). Amongst B6-*Lyst* and D2.*Lyst* mice, there was no difference in mean latency to fall from the accelerating rotarod up to 14 months of age. However, after 16 months of age, D2.*Lyst* exhibited reduced latencies compared to B6-*Lyst* mice (*p* = 4.4E^−2^, Bonferroni corrected *p*-value using a two-tailed Student’s *t-*test with two comparisons; Fig. 5a). Both strains showed general trends of increasing latency with successive trials (Fig. 5b). Within strains, latency to fall remained constant with advancing age in B6-*Lyst*, but decreased with age in D2.*Lyst* (*p* = 4.6E^−3^, Linear Regression Analysis with ANOVA; Fig. 5c,d). Among studies to identify factors influencing rotarod performance, some have identified that body weight can influence performance^38,39^. Body weight of D2.*Lyst* mice was significantly less than B6-*Lyst* mice at ages over 12 months of age (Fig. 5e), but latency to fall was not significantly correlated with body weight in either strain (B6-*Lyst*: adjusted *R*^*2*^ = 1.6E^−2^, *p* = 2.2E^−1^; D2.*Lyst*: adjusted *R*^*2*^ = 4.5E^−3^, *p* = 3.6E^−1^, Linear Regression Analysis with ANOVA).

### Quantitative assessments of cerebellar anatomy

**Figure 6:**
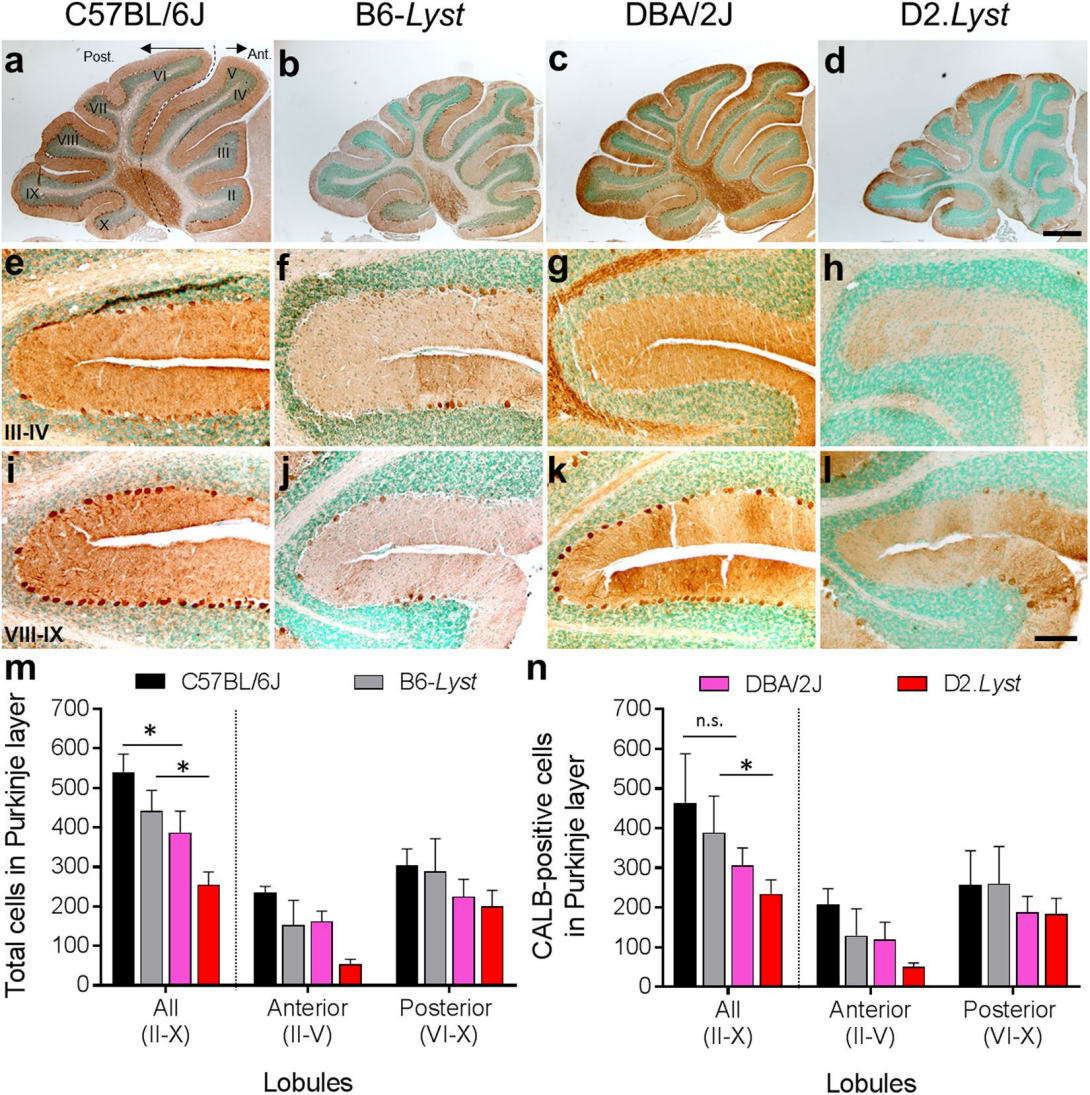
Cerebellar Purkinje cell loss associated with *Lyst* mutation is exacerbated on the DBA/2J compared to the C57BL/6J genetic background. Quantitative analyses of Purkinje cells from histological specimens of cerebella from two different inbred strains of age-matched (17–20 months) mice homozygous for either the wild-type (C57BL/6J and DBA/2J) or mutant *Lyst* allele (B6-*Lyst* and D2.*Lyst*). Representative images from position-matched mid-sagittal cerebellar sections immunohistochemically labeled with an anti-calbindin (CALB^+^, marker of Purkinje cells, brown staining) antibody and counterstained with methyl green (stains nuclei green) of: (**a**–**d**) whole cerebella, (**e**–**h**) anterior fissure between lobules III–IV, and (**i**–**l**) posterior fissure between lobules VIII-IX. Note differences in the density of dendritic branching and number of cells with CALB^+^ immunoreactivity between strains. Images in each column were collected from the same cerebellar specimen and presented in lower (top row) and higher (bottom two rows) magnification. Scale bars = 500 µm (**a**–**d**) and 100 µm (**e**–**l**). Graphs showing counts of: (**m**) total cells (sum of CALB^+^ cells plus cells with methyl green stained nuclei alone) and (**n**) presumptive Purkinje cells (CALB^+^ cells) detected in the Purkinje layer extending along the anterior (lobules II–V) and posterior (lobules II–V) zones of cerebellum. Each bar represents mean cell count ± SD from 3 histological sections of cerebella per mouse for (*n* = mice) of each strain (*n* = 3, C57BL/6J; *n* = 5, B6-*Lyst*; *n* = 3, DBA/2J; and *n* = 5, D2.*Lyst*). (*) represents a Bonferroni corrected *p* < 0.05 using a two-tailed Student’s *t*-test.

In order to better ascertain the nature of the Purkinje cell deficits in these strains, we performed quantitative analyses of Purkinje cell number in aged mice (17–20 months) homozygous for the mutant or wild-type *Lyst* allele in the context of both the B6 and D2 backgrounds (Fig. 6). In these analyses, cerebellar specimens were immunohistochemically labeled with an antibody against calbindin (CALB), a cell specific marker of Purkinje neurons in cerebellum^40^. As an additional assay to differentiate a loss of cells versus a potential loss of immunoreactivity, specimens were also stained with methyl green, a nuclear stain. In qualitative comparisons (Fig. 6a–l), *Lyst* mutation was associated with loss of Purkinje cells and decreased CALB-immunostaining for cell bodies and their processes, which were more pronounced in D2 than B6 and the anterior compared to posterior cerebellum. In comparing the quantified results of these two assays (Fig. 6m,n), a common pattern of cell loss was suggested in both—influenced by genetic background (D2 > B6), *Lyst* genotype (mutant > wild-type), and anatomic position (anterior > posterior). The strain dependent influences were largely supported by statistical comparisons (mean ± SD for *total cells*: 387 ± 54 for D2 vs. 540 ± 45 for B6, *p* = 4.0E^−2^; 441 ± 53 for B6-*Lyst* vs. 255 ± 32 for D2.*Lyst*, *p* = 3.0E^−4^; *CALB*^+^ *cells*: 234 ± 36 for D2.*Lyst* vs. 389 ± 92 for B6-*Lyst*, *p* = 1.6E^−2^), however one comparison failed to meet criteria for statistical significance (*CALB*^+^ *cells*: 307 ± 44 for D2 vs. 464 ± 123 for B6; *p* = 2.0E^−1^; Bonferroni corrected *p*-value using a two-tailed Student’s *t-*test with two comparisons).

Statistical significance was also assessed using a Poisson mixed effect model, in which the predictors were *Lyst* genotype and genetic background; the interaction of genotype with background were not included in this analysis because it was non-significant. This model was selected because there were two cell counts for each individual mouse, one for the anterior and one for the posterior cerebellum. In this analysis, both *Lyst* genotype (*p* = 1.8E^−2^) and genetic background (*p* = 4.58E^−7^) were determined to significantly influence the number of CALB^+^ cells in the Purkinje layer. To assess the effect that genotype and background each had on counts of CALB^+^ cells between the anterior and posterior regions of cerebellum, we conducted a linear regression in which the response was the difference of the log-transformed cell count between the two regions and the predictors were *Lyst* genotype and background. In this analysis, genotype was significant (*p* = 3.0E^−3^) but background was not (*p* = 8.2E^−2^). These results indicate the Purkinje cell loss associated with *Lyst* mutation disproportionately affects the anterior compared to posterior cerebellum, and is more severe on the D2 compared to the B6 background. Furthermore, the severe cell loss in D2.*Lyst* compared to B6-*Lyst* mice is approximately the same as the cell loss in D2 compared to B6—i.e., the particularly severe phenotype we have observed in D2.*Lyst* mice again appears to be the result of a predominantly additive effect.

### Bioinformatic identification and validation of candidate genes responsible for strain-dependent phenotypes

In order to identify candidate genes potentially contributing to the strain-dependent differences in cerebellar phenotypes of *Lyst*-mutants, we utilized publicly available data in two distinct bioinformatic-driven strategies, and subsequently tested leading candidates directly using RT-qPCR.

**Figure 7:**
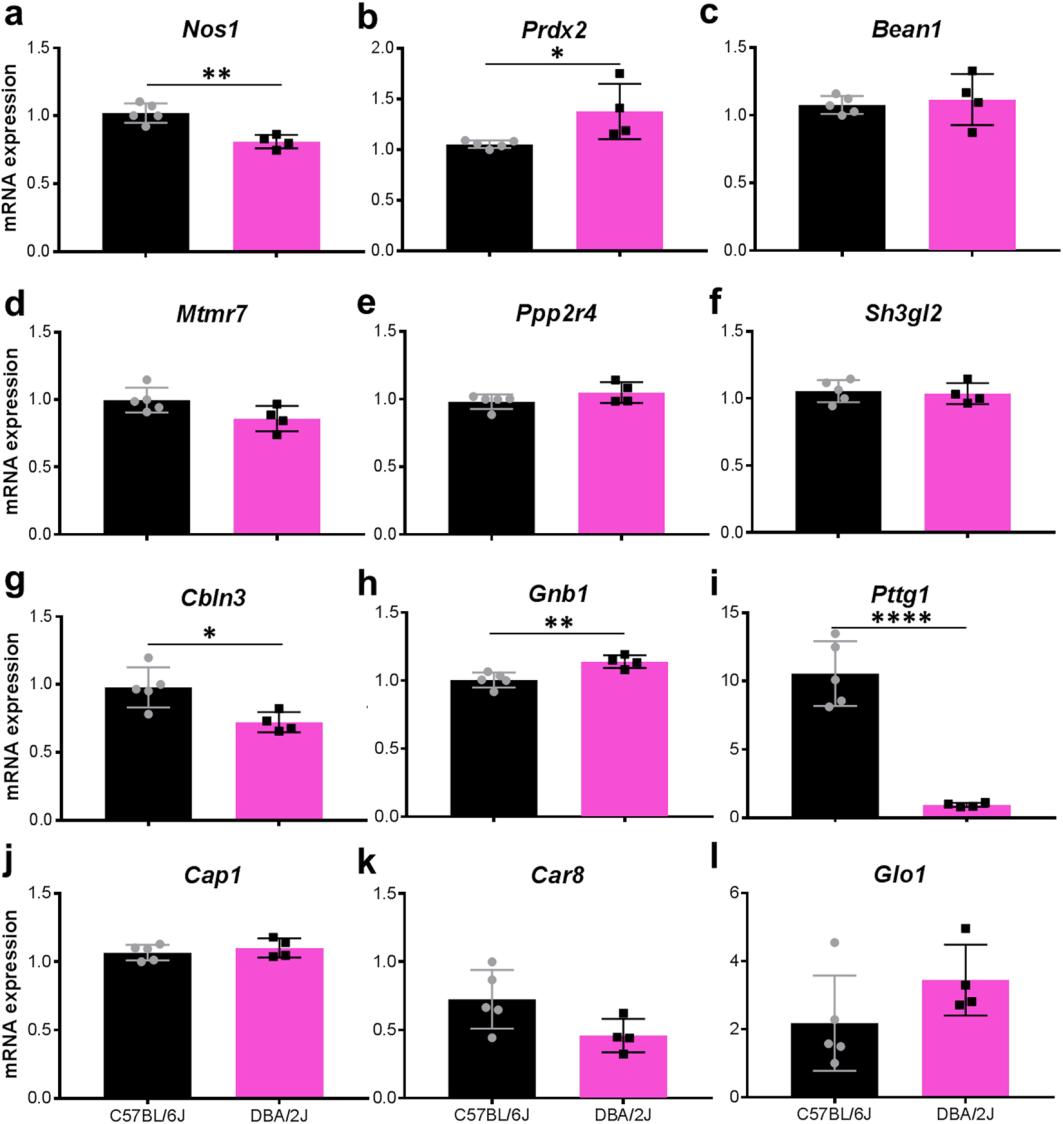
Differential expression of selected candidate genes responsible for exacerbating cerebellar phenotypes of mutant-*Lyst* mice on the DBA/2J compared to C57BL/6J genetic background. (**a**–**l**) Graphs showing mean relative normalized expression levels of the indicated mRNAs in the cerebellum, normalized to the level of *Actb*. Bars represent mean ± SD; unpaired two-tailed Student’s *t*-test analysis: *p < 0.05, **p < 0.01, ***p < 0.001 for *n* = 5 mice (C57BL/6J, black bars) and 4 mice (DBA/2J, pink bars).

In our first strategy, we aimed to identify robust candidates that had known roles in cerebellar physiology, that contained DNA variants likely to be deleterious in one of the strains, and/or that had altered RNA transcript levels between the strains—ideally all three. Because the approach relied on pre-existing publicly deposited datasets, there were compromises in the ages and sources of data, but 466 unique genes were nonetheless identified which met these criteria to various degrees. Of these, 190 were identified from a previously published microarray study on the cerebellum of B6 and D2 mice at postnatal day 6 (representing the top 250 probes identified from GSE60437; Supplemental Table 1). Adding to this, 283 were identified by searching the Purkinje cell transcriptome (defined by gene expression data of cerebellar Purkinje cells from 8-week-old B6 mice; GSE26749) for genes with variants likely to be deleterious (defined by splice variants, stop-lost and stop-gained variants identified by the Mouse Genomes Project^41,42^; Supplemental Table 2). Seven genes were in common to both lists (*Ccndbp1*, *Mrpl55*, *Msi*2, *Polr1a*, *Prkag*2, *Rhobtb3*, and *Zfp27*), but to our knowledge, none of these have striking links to cerebellar physiology or disease. Thus, we reverted to considering the combined list of all 466 genes and subjectively selected 6 candidates for independent testing of expression by RT-qPCR. Candidates included were *Bean1*, which contributes to spinocerebellar ataxia^43^; *Sh3gl2*, which functions as a binding partner to other proteins causing spinocerebellar ataxia^44,45^; *Nos1*, *Mtmr7*, and *Ppp2r4*, which are known to influence cell death^46–48^; and *Prdx2*, which is known to confer protection from oxidative stress^49,50^. Using RNA extracted from the cerebellum of age-matched (8–10 weeks of age) adult B6 or D2 mice (Fig. 7a–f), we detected statistically significant changes in expression in two genes: *Nos1* (B6 > D2) and *Prxd2* (B6 < D2; *p* ≤ 0.04 for both genes; Fig. 7a,b). We found no strain-specific differences in cerebellar expression of *Bean1*, *Mtmr7*, *Ppp2r4*, or *Sh3gl2* (Fig. 7c–f).

The leading candidate from this approach was *Nos1*. By RT-qPCR, we confirmed *Nos1* to have the same directionality of expression between strains and detected a modest, but statistically significant, 1.27-fold reduction in D2 relative to B6 mice. *Nos1* was predicted to have a splice donor variant in a UCSC-annotated long non-coding RNA transcript that could affect transcript stability. The expression of *Prdx2* detected in our RT-qPCR validation assay with adult mice was in the opposite direction of that predicted by the public microarray data from mice at age P6. However, our result is consistent with expression data for the cerebellum of adult B6 and D2 mice accessible via the GeneNetwork database (see below). In sum, this approach identified many genes that may influence the cerebellum of B6 or D2 mice, with one (*Nos1*) that met more criteria than the others tested, but none that met our full criteria for a robust candidate.

In our second strategy, we aimed to identify cis-acting expression quantitative trait loci (cis eQTL), which can result in biologically-relevant changes responsible for complex traits^51^. To achieve this, we used cerebellar mRNA microarray expression data via the publicly accessible GeneNetwork website to generate a list of significant cis eQTL between B6 and D2 mice. We identified 83 expression probes, corresponding to 71 genes, with a likelihood ratio statistic (LRS) greater than 15 (Table 1; in order of decreasing LRS). To promote stringency, we additionally filtered for genes represented by two or more probes that exhibited the same directionality of expression between the B6 and D2 strains. From this reduced list, 6 genes having the greatest LRS (*Gnb1, Cap1, Glo1, Car8, Cbln3*, and *Pttg1*) were selected for independent testing of expression in the adult cerebellum between strains (Fig. 7g–l). By RT-qPCR, all 6 candidate genes were confirmed to be differentially expressed and with the predicted directionality of expression between strains, 3 of which were statistically significant. Expression of *Cbln3* and *Pttg1* transcripts was detected at significantly lower levels, whereas *Gnb1* was detected at significantly higher levels in D2 cerebella compared to B6 (*p* ≤ 0.02 for all three genes; Fig. 7g–i). According to the Mouse Genomes Project^41,42^, *Cap1* has one missense variant, *Pttg1* has three missense variants, *Glo1* is duplicated in D2 relative to B6, whereas *Gnb1*, *Car8*, and *Cbln3* contain no variants predicted to alter protein sequence.

**Table 1:** List of cis-acting expression quantitative trait loci detected in the adult mouse cerebella.

Probe	Symbol	Gene Location (Chr: Mb)	Mean Expr.	Max LRS	Max LRS Location (Chr:Mb)
1452705_at_A	Pdxdc1	Chr16: 13.834279	8.8	163.8	Chr16: 13.142340
1417432_a_at_A	Gnb1	Chr4: 155.557168	9.7	150.8	Chr4: 155.502574
1431708_a_at_B	C87436	Chr6: 86.421524	8.6	141.3	Chr6: 85.817555
1417461_at_A	Cap1	Chr4: 122.859245	10.0	141.1	Chr4: 122.859565
1438390_s_at_A	Pttg1	Chr11: 43.420325	12.0	136.5	Chr11: 43.030624
1430979_a_at_A	Prdx2	Chr8: 84.974348	9.0	133.4	Chr8: 83.183208
1417462_at_A	Cap1	Chr4: 122.860065	8.8	127.3	Chr4: 122.859565
1451457_at_A	Sc5d	Chr9: 42.254792	7.5	121.2	Chr9: 42.062548
1437478_s_at_B	Efhd2	Chr4: 141.858490	11.6	114.6	Chr4: 140.610378
1436240_at_B	Tra2a	Chr6: 49.256479	8.2	113.9	Chr6: 48.979304
1446540_at_B	Kirrel3	Chr9: 34.711363	10.7	113.4	Chr9: 34.576651
1429628_at_B	KIAA0408	Chr10: 29.197127	9.4	110.3	Chr10: 28.781312
1431473_at_B	Kirrel3	Chr9: 34.752667	7.7	110.2	Chr9: 34.576651
1424105_a_at_A	Pttg1	Chr11: 43.422964	8.2	108.5	Chr11: 43.030624
1455136_at_A	Atp1a2	Chr1: 172.271794	10.5	108.2	Chr1: 172.271794
1424109_a_at_A	Glo1	Chr17: 30.594167	12.9	107.2	Chr17: 27.848224
1449578_at_A	Supt16h	Chr14: 52.175266	8.0	106.1	Chr14: 49.789692
1444128_at_B	Arhgap26	Chr18: 39.372011	8.5	102.1	Chr18: 39.042559
1454696_at_A	Gnb1	Chr4: 155.558771	13.4	101.2	Chr4: 155.502574
1450712_at_A	Kcnj9	Chr1: 172.321699	10.0	100	Chr1: 172.271794
1456257_at_B	Fam126b	Chr1: 58.524799	9.9	99.5	Chr1: 58.116419
1457973_at_B	2310043D08Rik	Chr14: 93.827703	9.6	95.2	Chr14: 93.711204
1457904_at_B	Car8	Chr4: 8.141530	9.9	94.6	Chr4: 6.893555
1420286_at_A	Cacna1a	Chr8: 84.415028	9.1	93.5	Chr8: 83.183208
1428083_at_A	2310043N10Rik	Chr19: 5.842572	9.9	93.1	Chr19: 4.845967
1424454_at_A	Tmem87a	Chr2: 120.355405	9.8	90.4	Chr2: 118.881030
1451240_a_at_A	Glo1	Chr17: 30.592883	8.7	90.1	Chr17: 27.848224
1425614_x_at_A	H2-Q2	Chr17: 35.395547	8.6	88.1	Chr17: 33.110219
1426766_at_A	Nlrp1a	Chr11: 71.032066	10.6	87.6	Chr11: 70.557664
1455445_at_B	Cbln3	Chr14: 55.878941	14.2	87.2	Chr14: 55.622689
1437708_x_at_A	Vamp3	Chr4: 151.047315	10.3	86.2	Chr4: 150.309420
1423150_at_A	Scg5	Chr2: 113.776720	12.8	86.1	Chr2: 112.284850
1434292_at_A	Snhg11	Chr2: 158.385574	11.8	85.7	Chr2: 157.995222
1435579_at_B	AK138571	Chr4: 42.710724	8.5	85.4	Chr4: 41.025573
1451411_at_A	Gprc5b	Chr7: 118.973674	10.5	84.6	Chr7: 119.117526
1441404_at_B	Pafah1b1	Chr11: 74.706619	8.1	84.4	Chr11: 74.110266
1452544_x_at_A	EG630499	Chr17: 33.974942	9.4	84.1	Chr17: 33.110219
1438059_at_B	Ctxn3	Chr18: 57.477539	7.4	83.6	Chr18: 57.293889
1460045_at_B	Cdh7	Chr1: 109.996442	8.9	83.5	Chr1: 109.064274
1437308_s_at_A	F2r	Chr13: 95.601835	7.6	81.4	Chr13: 95.700999
1455645_at_B	Mybpc1	Chr10: 88.518361	8.2	79.8	Chr10: 87.643398
1457324_at_B	Sox12	Chr4: 42.017173	8.8	79.8	Chr4: 41.025573
1439843_at_B	Camk4	Chr18: 33.195287	12.9	79.1	Chr18: 31.556238
1436763_a_at_A	Klf9	Chr19: 23.164957	9.3	78.8	Chr19: 23.165770
1432198_at_B	Prune2	Chr19: 17.222807	12.5	76.6	Chr19: 16.881460
1455806_x_at_A	Ndufa12	Chr10: 94.220784	13.1	76.5	Chr10: 91.919545
1434893_at_A	Atp1a2	Chr1: 172.273211	12.2	75.7	Chr1: 172.271794
1428370_at_B	C12orf76	Chr5: 114.808310	11.0	75.7	Chr5: 115.147874
1415978_at_A	Tubb3	Chr8: 123.421601	9.8	73.3	Chr8: 123.700192
1449018_at_A	Pfn1	Chr11: 70.652008	12.0	72.9	Chr11: 70.557664
1422911_at_A	Cbln3	Chr14: 55.881770	12.8	70.8	Chr14: 55.622689
1447939_a_at_B	Ccl21	Chr4: 42.089209	10.1	70.3	Chr4: 41.025573
1429685_at_B	Gabrb2	Chr11: 42.630135	11.2	67.8	Chr11: 42.371999
1424958_at_A	Car8	Chr4: 8.143491	11.9	65.7	Chr4: 6.893555
1436713_s_at_A	Meg3	Chr12: 109.544750	9.2	65.2	Chr12: 107.898571
1438511_a_at_A	Rgcc	Chr14: 79.288778	7.7	64.7	Chr14: 78.383350
1435129_at_A	Ptp4a2	Chr4: 129.839203	10.1	63.4	Chr4: 129.572170
1447937_a_at_B	CCL_complex	Chr4: 41.810128	10.0	63.1	Chr4: 41.025573
1456341_a_at_A	Klf9	Chr19: 23.165355	12.9	61.9	Chr19: 23.165770
1444147_at_B	Pcsk2	Chr2: 143.579595	7.7	60.4	Chr2: 142.704064
1424877_a_at_A	Alad	Chr4: 62.509266	9.0	60.2	Chr4: 61.857952
1417151_a_at_A	Ntsr2	Chr12: 16.658423	11.7	59.5	Chr12: 16.828469
1437774_at_B	Spint1	Chr2: 119.602619	10.9	59.5	Chr2: 118.881030
1455316_x_at_A	LINE-1	Chr13: 66.715420	18.2	58	Chr13: 64.085711
1447938_at_B	CCL_complex	Chr4: 42.468451	10.3	56.8	Chr4: 41.025573
1448300_at_A	Mgst3	Chr1: 167.372516	12.5	55.7	Chr1: 166.983006
1436766_at_A	Luc7l2	Chr6: 38.568986	8.6	52	Chr6: 38.129136
1448715_x_at_A	NM_010490	Chr13: 66.715420	16.9	50.1	Chr13: 64.085711
1459731_at_B	BE996194	Chr11: 88.701963	8.8	47.3	Chr11: 88.661619
1423506_a_at_A	Nnat	Chr2: 157.561887	12.6	47	Chr2: 156.863893
1437311_at_B	Snhg11	Chr2: 158.381003	10.9	46.2	Chr2: 157.995222
1441937_s_at_B	Pink1	Chr4: 138.313503	9.7	43.4	Chr4: 138.596456
1431225_at_B	Sox11	Chr12: 27.337989	7.5	43.3	Chr12: 26.307604
1452975_at_B	Agxt2l1	Chr3: 130.635223	9.8	42.4	Chr3: 129.825025
1438663_at_B	Prrc2c	Chr1: 162.672315	10.6	34	Chr1: 160.285087
1418282_x_at_A	Serpina1b	Chr12: 103.728347	7.7	33.3	Chr12: 103.031681
1417600_at_A	Slc15a2	Chr16: 36.750199	9.1	32.9	Chr16: 39.118020
1419423_at_A	Stab2	Chr10: 86.841213	9.0	31.8	Chr10: 86.451679
1455892_x_at_A	Ccdc21	Chr4: 134.164032	14.4	30.2	Chr4: 132.854094
1431213_a_at_A	LOC433762	Chr4: 133.098515	13.7	29.2	Chr4: 132.854094
1431214_at_A	LOC433762	Chr4: 133.098690	12.0	29	Chr4: 132.854094
1442019_at_B	Gas7	Chr11: 67.687197	12.8	24.5	Chr11: 68.147545
1423256_a_at_A	Atp6v1g1	Chr4: 63.550589	10.7	15.1	Chr4: 63.685588

Footnotes. For column headers: Max Expr. = maximum expression, Max LRS = maximum likelihood ratio statistic.

## Discussion

Previously, we have shown that D2.*Lyst* mice exhibit a tremor phenotype that is genetic background dependent and is associated with a loss of cerebellar Purkinje cells^26^. Here, these results identify three factors that impart a significant influence on the severity of CHS-related neurologic deficits. Genetic background, *Lyst* genotype, and age were each identified as factors that impart a significant influence on the severity of CHS-associated neurologic deficits, culminating in more severe phenotypes in aged D2.*Lyst* compared to B6-*Lyst* mice. We have also defined that *Lyst* mutation preferentially affects the loss of Purkinje cells in the anterior lobules. The assays also establish a baseline, whereby further mechanistic work studying CHS-associated neurologic deficits could be carried out using mice, including the identification of modifier genes.

Phenotype-driven approaches have the advantage of being agnostic (not depending on pre-existing knowledge) and can identify novel naturally occurring alleles with physiological effects^52–57^. The key feature that empowers phenotype-driven genetics is the existence of robust assays that differentiate distinct genetic contexts. Our current results identify an ordinal phenotypic assay, the CPSS for cerebellar ataxia developed by Guyenet *et al*.^27^, which could be utilized in phenotype-driven genetic approaches to map modifiers relevant to CHS-associated neurologic disease. Advantages of using the CPSS are that it can differentiate phenotypes in mice prior to 6 months of age, is a rapid assay readily adaptable to high-throughput screens, and does not require specialized equipment. One caveat of using this assay is that even among age-matched mice that are genetically identical, individual-to-individual variation in phenotypic response were readily apparent. Therefore, accurate genotype:phenotype associations might require large numbers of mice. Although additional experiments would be required to stringently test *Lyst*-dependency, our results suggest that the strain differences are likely to be additive—hinting that other models of cerebellar disease exhibiting undetectable or mild phenotypes on the widely used B6 background may also be made more severe if studied on the D2 background. Given the rapid advances allowing new mouse models to be made on a wide variety of inbred backgrounds using CRISPR/Cas9 technology, this is an option that may be worthy of broad consideration by the field.

These experiments indicate that the accelerating rotarod assay could also be utilized in phenotype-driven genetic approaches using quantitative data to map modifiers relevant to CHS-associated neurologic disease. The main advantage of using this assay is that it yields truly quantitative data appropriate for traditional quantitative trait analyses. Potential caveats of using this assay include: (1) As with the CPSS, the accelerating rotarod assay would also be influenced by individual-to-individual variation that may potentially confound genotype:phenotype correlations. (2) Visual function is a biological factor potentially confounding the rotarod assay and D2 mice can develop an age-related form of glaucoma^37,58^. (3) Mice would need to be aged to approximately 16 months of age. (4) The rotarod assay is comparatively time-consuming. Because we did not test purely inbred B6 and D2 mice with accelerating rotarod, the results do not address how differences between the B6 and D2 strains might be additive, as they were in the CPSS for cerebellar ataxia.

Together, our results indicate that the CPSS provides greater ability to differentiate *Lyst*-dependent neurologic phenotypes than the accelerating rotarod assay. Results from both assays were consistent with those from our quantitative analyses of cerebellar Purkinje cells—i.e., the strains with worse performance had fewer Purkinje cells in the cerebellum. It would be interesting to compare correlations between CPSS scores with cerebellar Purkinje cell number in individual mice, but we could not attempt this here because our analyses utilized different cohorts of mice. We suspect that the greater ability of the CPSS to distinguish strain differences in our study relates to the fact that it integrates multiple metrics for anatomic (kyphosis), behavioral (hind limb clasping), and motor coordination (ledge and gait tasks) phenotypes, whereas the rotarod assay is based on a single task of motor coordination.

Our results revealed that *Lyst* genotype significantly influenced Purkinje cell number. This Purkinje cell deficit was detected primarily in the anterior lobules compared to those in the posterior cerebellum, a result consistent with other reports that describe patterned losses of these neurons in mouse models of cerebellar disease^59–61^. However, some limitations to these analyses merit note. First, the influence of genetic background fell short of traditional cut-offs for statistical significance in some analyses. Second, our analyses were performed on cerebellar specimens from limited numbers of mice at a single point in time at advanced age. In future work, it would be worthwhile to quantify Purkinje cells from more mice, and at younger ages, to determine whether this assay might also have utility in genetic experiments to identify the strain dependent modifier genes implied to exist by our current results.

This work identified genes that might contribute to the strain-dependent differences we have observed, but at a minimum, clearly have overt mutations or differential levels of expression in the cerebellum between B6 and D2 mice. Candidates identified by our approaches, which we were able to independently confirm changes in cerebellar transcript abundance for, are *Nos1*, *Prdx2*, *Cbln3*, *Gnb1*, and *Pttg1*. Of these, *Pttg1* is of particular interest. Strain-dependent differences in *Pttg1* expression have previously been discovered through a study of recombinant inbred strains of mice^62^. Across a broad range of inbred strains, B6 is an outlier, displaying an aberrant *elevated* level of expression, which was previously shown to be associated with a seven nucleotide deletion in the *Pttg1* promoter. The *Pttg1* gene, whose full name is “pituitary tumor-transforming gene 1” encodes a multifunctional protein that can bind p53 and inhibit the ability of p53 to induce cell death^63^. Because p53 has been implicated in cerebellar development^64,65^ and maintenance^66^ in multiple ways, it is feasible—albeit speculative—that B6 mice with elevated *Pttg1* expression could have less p53-mediated cell death and a more robust Purkinje cell population.

Two additional candidates of note are *Glo1* and *Cbln3*. The *Glo1* gene, encoding “glyoxalase 1,” is known to be duplicated in D2 and other strains of mice^67^. Although we were unable to independently confirm a statistically significant up-regulation of *Glo1* in D2 compared to B6, a trend of approximately 2-fold expression was readily observable. GLO1 is important to the cerebellum^68^, but an increase in GLO1 levels would be predicted to protect against oxidative stress and enhance cell survival in the D2 cerebellum—which is opposite to our RT-qPCR result. *Cbln3* is a member of the cerebellin gene family, which encode secreted proteins that act as *trans*-synaptic cell adhesion molecules. CBLN3 is restricted to the cerebellum, where it binds to CBLN1. Mice lacking CBLN1 exhibit motor defects such as ataxia and deficits in rotarod performance^69,70^, but mice lacking CBLN3 have no observable phenotypes^71^. Thus, the strain-specific differences in *Glo1* and *Cbln3* expression we have observed could have relevance to the cerebellum through many different pathways, but they do not currently lend themselves to simplistic hypotheses with respect to the phenotypes we are primarily interested in.

It is important to not over interpret our study of candidates potentially influencing the cerebellum. We have presented large amounts of information in Table 1 and in the Supplemental Tables, which we believe could be independently useful for other researchers studying the cerebellum, but genetic studies are needed to stringently identify the implied genetic modifier(s). There were also some limitations to the analyses. First, some classes of mutations were not included in the prioritization of our first approach. Thus, the list of candidates identified is not exhaustive. Second, some of the genetic variations appear to occur in transcripts that are not the predominant one, which we suspect contributed to the poor confirmation rate of our first approach. Third, re-sequencing of genomic DNA to confirm variants (listed in Supplemental Table 2) was not performed. Finally, our analyses included data from mice of multiple ages (8 weeks, GSE26749; post-natal day 6, GSE60437; “adult”, GeneNetwork; 8–10 weeks, RT-qPCR), but none that were extensively aged. The confounding potential for these mismatches were particularly evident in our first approach, in which statistically significant changes of *Prdx2* were detected in opposite directions in the microarray data from post-natal day 6 mice versus RT-qPCR data from mice 8–10 weeks old.

## Methods

### Animal husbandry

C57BL/6J (B6), C57BL/6J-*Lyst*^*bg-J*^/J (abbreviated throughout as B6-*Lyst*), and DBA/2J (D2) mice were obtained and remain commercially available at The Jackson Laboratory (Bar Harbor, Maine). Congenic mice homozygous for the *Lyst*^*bg-J*^ mutation (D2.B6-*Lyst*^*bg-J*^/Andm; abbreviated throughout as D2.*Lyst*) were generated through reiterative backcrossing as previously described^26^, and maintained as a homozygous stock at the University of Iowa. All experiments with *Lyst-*mutant mice utilized individuals homozygous for the *bg-J* mutation. Mice were housed at the University of Iowa Research Animal Facility, maintained on a 4% fat NIH 31 diet provided *ad libitum*, and kept in cages containing dry bedding (Cellu-dri; Shepherd Specialty Papers). The environment was kept at 21 °C with a 12-hour light:12-hour dark cycle. During aging, individual mice exhibiting decreases in body weight ≥ 20% relative to the corresponding strain average at 12 months of age were omitted from the study. All animals were treated in accordance with the Association for Research in Vision and Ophthalmology Statement for the Use of Animals in Ophthalmic and Vision Research. All experimental protocols were approved by the Animal Care and Use Committee of the University of Iowa.

### Tissue preparation, immunohistochemistry, and microscopy

Mice were euthanized by CO_2_ inhalation; brains were removed and fixed in 4% para-formaldehyde (PFA) in phosphate buffered saline (PBS) for four hours at 4 °C. Cerebella were dissected, bisected into halves, with one half processed for embedding in frozen optimal cutting temperature medium (OCT, Tissue-Tek OCT Compound, Sakura) and the other in paraffin. For processing in OCT, fixed specimens were cryoprotected with increasing concentrations of sucrose (5% to 30%), embedded in a mixture (2:1) of 30% sucrose:OCT-medium, flash frozen, and stored at −80 °C. For processing in paraffin, fixed specimens underwent standard paraffin embedding, including dehydration in graded ethanol (70–100%), clearing in xylenes, and embedding in molten paraffin.

All immunohistochemistry was performed using a polyclonal antibody (anti-CALBINDIN D-28K, AB1778, Millipore) on position-matched mid sagittal histological sections of mouse cerebella (each section included lobules II - X) followed by two distinct labeling techniques, each used for either (1) qualitative or (2) quantitative analyses, as described below.

(1) For qualitative immunohistochemical analyses, histologic sections were cut from frozen OCT embedded cerebellar specimens using a cryostat (Cryocut 1800, Reichert Jung, Ametek), mounted to glass slides, and stored at −20 °C. Frozen sections were post-fixed in 2% PFA, rinsed in PBS, blocked with 5% normal donkey serum, 0.3% Triton X-100, and 0.01% sodium azide in PBS, incubated with primary antibody diluted (1:1,000) in blocking solution for 18 hours at 4 °C, incubated in a donkey anti-goat secondary IgG antibody (Alexa Fluor 488, 1:500, Life Technologies), mounted with Vectashield medium with DAPI (Vector Laboratories), and cover-slipped. Cerebellar sections were imaged at anatomically-matched locations in the cerebella at total magnifications of 100X and 200X using a light microscope (BX52; Olympus) equipped with a digital camera (DP72; Olympus). All immunofluorescence images were collected using identical camera settings and were enhanced using identical settings in Adobe Photoshop imaging software. Following imaging, coverslips were soaked off in distilled water and slides were stained with hematoxylin and eosin (H&E) using standard methodology, dehydrated in a series of graded ethanol (80% to 100%), cleared with xylenes, and cover-slipped with mounting medium (MM24, Leica Microsystems). Cerebellar sections were re-imaged at the same anatomic position, magnifications, and light microscope, as described previously. All images were collected using identical camera settings and enhanced using identical settings in Adobe Photoshop imaging software.

(2) For quantitative histological analyses, histologic sections were cut from cerebellar specimens embedded in either OCT (as described in #1 above) or paraffin using a microtome (Reichert Jung Biocut 2030, Ametek), and mounted on glass slides (Superfrost Plus Slides, Fischer). Paraffin sections were exclusively deparaffinized by incubation at 60 °C and rinsing in xylenes, rehydrated in decreasing concentrations of ethanol in distilled water, and incubated at 95 °C in 10 mM citrate buffer using an oven (Pelco Biowave, Ted Pella) for epitope retrieval. All mounted sections were briefly post-fixed in 2% PFA, permeabilized in 0.15% Triton-X100 in PBS for 10 minutes, rinsed (and all subsequent rinses) in 0.1% Tween-20 in PBS, quenched for endogenous peroxidase activity for 15 minutes in 3% hydrogen peroxide in PBS, blocked in 2.5% normal horse serum, incubated in primary antibody (diluted 1:500 in antibody diluent #IW1000; IHC World) for 18 hours at 4 °C, treated with a horse radish peroxidase based detection system (ImmPRESS HRP anti-Rabbit Reagent kit; MP-7401-50; Vector Laboratories) and developed (DAB Substrate Kit, ab64238, Abcam) following their respective included protocols, counterstained with methyl green, dehydrated in 95% and 100% ethanol, cleared in xylenes, and cover-slipped with MM24 mounting medium. Representative images of cerebellar sections were collected in identical fashion as described in part (1) above.

### Quantification of cerebellar Purkinje cells

The quantification and presentation of Purkinje cell count data were performed using a methodology similar that which has been previously described^59^. To describe in more detail, stained histologic sections of cerebella (*n* = 3 sections from each mouse; as described in part 2 above), were collected from mice homozygous for the *Lyst* mutation (*n* = 5 mice at 17–19 months of age for both B6-*Lyst* and D2.*Lyst* strains; *n* = 15 sections per mutant strain) and wild-type allele (*n* = 3 mice at 20 months of age for both B6 and D2 strains; *n* = 9 sections per wild-type strain)) were imaged using a digital slide scanner (Aperio Scanscope CS; Leica Biosystems) at 20X magnification and 7000 × 7000 px resolution. For analyses, a blinded investigator performed manual counts of cells along the Purkinje cell layer spanning lobules II through X of each cerebellum from digital images using the cell counter tool in Image-J^72^ image analysis software. Cells in the Purkinje layer were counted and organized in two categories: (1) “total cells in the Purkinje layer,” which was the sum of cells with methyl green nuclear staining (methyl green^+^) alone plus cells positive for both methyl green^+^ and CALB immunostaining (CALB^+^) and (2) “CALB^+^ cells in the Purkinje layer,” which were composed of cells positive for both (CALB^+^ and methyl green^+^). Cell counts from each of three sections per cerebellar specimen per mouse were averaged and then combined with other individuals of the same strain to calculate the mean cell count for each strain. Data were expressed as mean cell count ± SD for each strain and organized by total counts and subdivided by anatomical position (anterior lobules II-V versus posterior lobules VI-X) within the cerebellum. Statistical comparisons were made using: a two-tailed Student’s *t*-test with Bonferroni correction for two comparisons to test for differences according to genetic background (B6 vs. D2) and *Lyst* genotype (B6-*Lyst* vs. D2.*Lyst*) within each of the two count categories. Poisson mixed effects modeling was performed using the R statistical computing environment^73^.

### Neurobehavioral assessments and CPSS for cerebellar ataxia

Mice were subjected to four distinct physical or behavioral tests and scored by three separate judges, of whom were masked to genotype, to assess phenotypic severity of ataxia, as described previously^27^. In brief, all three judges simultaneously scored (while masked to one another’s score and identity of each mouse) each mouse, based on its performance/condition in each of the following measures: (1) hind limb clasping^74^, (2) ledge test, (3) gait test, and (4) kyphosis^75^. Each measure is scored (0–3) and the combined scale of all four measures ranges from (0–12). Scoring data from individual tests (Fig. 4) and as a composite (scores from all four measures combined, Fig. 3) for each group of mice, which are organized by genotype and binned by age. Within each binned age group (in order as: 0–5.9, 6–11.9, 12–17.9, 18–30 months), the numbers of mice assessed are as follows: D2.*Lyst* (*n* = 30, 27, 39, 6 mice), B6-*Lyst* (*n* = 29, 13, 19, 8), DBA/2J (*n* = 22, 13, 4, 7), and B6 (*n* = 24, 19, 6, 3). Statistical comparisons were made using linear regression analyses and ANOVA using the R statistical computing environment^73^, to assess whether age, genetic background, and *Lyst* genotype imparted significant effects on composite scores; and two-tailed Student’s *t*-tests with a Bonferroni correction for multiple tests to assess differences according to genetic background (B6 vs. D2) and *Lyst* genotype (B6-*Lyst* vs. D2.*Lyst*) of mice.

### Accelerating rotarod assessments

Mice were tested for active performance on an accelerating rotarod apparatus (47600, Ugo Basile) as previously described^76^. In brief, mice were habituated to the rotarod for 240 seconds and subsequently tested in three trials (with at least 30 minutes rest between each trial) per day for four consecutive days. In each trial, the rotarod accelerated from speeds of 4–40 rpm over 240 seconds, and was then held constant at 40 rpm for the remainder of the trial (total duration of trial = 400 seconds). For each mouse, the time it took to fall off the rod (i.e. latency to fall) or cling to the rod without forward movement for two-consecutive rotations was recorded. Data are expressed as mean latency ± SEM (for each day, Fig. 5b) and as mean latency ± SEM (for all four days combined, Fig. 5a) for each group, which are organized by genetic background and binned by age as follows: B6-*Lyst* (5–7 months, *n* = 10 mice; 9–11 months, *n* = 5; 12–14 months, *n* = 7; 16–21 months, *n* = 5) and D2.*Lyst* (*n* = 10 at each binned age group). Data were omitted from study if any mouse showed presence of: overt injury (ex. fight wounds, dermatitis from excessive barbering, etc) or critically low body weight (≥20% loss) at or before the time of assay. Individual data points were excluded from analyses if deviated by ≥2 SD from the mean for that strain and age for each day of testing.

### Cerebellar gene expression and sequence queries

Data were collected from publicly available online resources and organized into three separate data tables. First, a previous analysis of gene expression in cerebellar Purkinje cells of B6 mice (8 weeks of age) using probe set (GPL1261) on the Affymetrix GeneChip Mouse Genome 430 2.0 Array platform was accessed using the NCBI Gene Expression Ombnibus (GEO) website^77^ to identify transcripts present in the cerebellum (GSE26749). In parallel, a list of all known genetic variants on the D2 genetic background, relative to the reference sequence of B6, was generated using the *query* tool on the Mouse Genomes Project^41,42^ tab at the Sanger Wellcome Trust Institute website^78^ with the following SNP/Indel search inclusion criteria: splice donor and acceptor variants, stop-lost and -gain variants, and frameshift variants across the length of chromosomes. This list of known genetic variants (*n* = 744) on the D2 genetic background, compared to the reference B6 background, was then cross-referenced to the probe set using Microsoft Access, and curated in table format, in order of increasing detection *p*-value using Microsoft Excel, to present a list of sequence variants in genes expressed in the cerebellum of D2 mice (*n* = 283; Supplemental Table 2).

Second, a previously published analysis of gene expression on cerebella from age-matched (postnatal day 6) B6 and D2 mice was accessed on the GEO website (GSE60437)^79^. Data were extracted, using the GEO2R analysis tool, to automatically generate a list of the most differentially expressed transcripts in cerebella between B6 and D2 mice (included 250 probes corresponding to 190 genes). Data were subsequently ranked in order of increasing *p*-value and organized into table format using Microsoft Excel (Supplemental Table 1).

Third, the GeneNetwork (GeneNetwork; 11/17/2018, RRIP:SCR_002388) website was used to identify cis eQTL in the cerebella of adult B6 and D2 mice in order to generate a list of potential modifiers responsible for the strain dependent phenotypes. A preliminary gene list was generated using the following search parameters: 1) Species: mouse (mm10), 2) Group: BXD RI Family, 3) Type: cerebellum mRNA, 4) Data Set: SJUT cerebellum mRNA M430 (Mar05) RMA, 5) Combined: cisLRS = (15 900 3) mean = (7 900) range = (5 900). This search identified 83 expression probes corresponding to 71 unique genes: that had an LRS greater than 15, mapped to within 3 Mb of the gene from which it is expressed, that were expressed above baseline, and showed a fold change of 5X or greater between B6 and D2 and subsequently organized into table format in order of decreasing LRS using Microsoft Excel (Table 1). This list of 71 genes was filtered down to 9 by retaining only those genes on the list represented by two or more probes with the same directionality of expression between B6 and D2. This list of 9 genes was then re-ordered by LRS and the 6 having the highest LRS were selected for independent testing of expression in the cerebellum of adult B6 and D2 mice (Fig. 7g–l).

### Tissue collection, RNA extraction, and quantitative real time-PCR

B6 (*n* = 5) and D2 (*n* = 4) female mice matched at ages between 8–10 weeks were deeply anesthetized with Ketamine/Xylazine (87.5 mg/kg/12.5 mg/kg) and euthanized by cervical dislocation. Immediately after euthanasia, the cerebellum was dissected and stored in RNAlater solution (Ambion, Applied Biosystems). Extraction of total RNA was performed using the RNeasy Plus Mini Kit (Qiagen Gmbh) following the manufacturer’s instructions. Lysis of tissue was performed with the TissueLyser LT (Qiagen Gmbh), using one steel ball at a speed of 50 Hz for 5 minutes. Traces of genomic DNA were removed using the DNA-free kit (Ambion, Applied Biosystems) following the manufacturer’s instructions. The concentration and quality of RNA were assessed using a Nanodrop 1000. The RNA was reverse transcribed using the iScript™ cDNA Synthesis Kit (Bio-Rad Laboratories Inc.) according to the manufacturer’s instructions. Predesigned TaqMan Gene Expression Assays (Applied Biosystems, Thermo Fisher) were used to determine the expression levels of the mRNAs of interest normalized to the levels of *Actb* mRNA with a RT-qPCR detection system (C1000 Thermal Cycler; Bio-Rad Laboratories). The composition of the reaction mixtures was: TaqMan Gene Expression Master Mix (1x), TaqMan Gene Expression Assay for the target mRNA (1x) and TaqMan Gene Expression Assay for reference mRNA (*Actb*, 0.75x), and RNA at a concentration of 0.512 ng/uL with exception of *Bean1*, which was at a concentration of 2.47 ng/uL. The following mRNA targets were assayed using following probes: *Gnb1* (Mm005150002_m1), *Cap1* (Mm00482950_m1), *Pttg1* (Mm00479224_m1), *Car8* (Mm00801469_m1), *Cbln3* (Mm00490772_m1), *Ppp2r4* (Mm00446807_m1), *Mtmr7* (Mm00457624_m1), *Sh3gl2* (Mm00490393_m1), *Nos1* (Mm01208059_m1), *Prdx2* (Mm04208213_g1), *Bean1* (Mm01250908_m1), *Actb* (Mm02619580_g1). PCR conditions were as follows: 50 °C for 2 minutes, 95 °C for 10 minutes followed by 40 × (95 °C for 15 seconds, 60 °C for 1 minute). Each experiment included 3 technical replicates of each RNA sample. Expression data were analyzed using Bio-Rad CFX Manager software, normalized to the levels of *Actb* mRNA, and expressed in terms of fold change relative to one sample randomly selected by the analysis software. Statistical analyses were performed in GraphPad Prism 7 graphing software using an unpaired two-tailed Student’s *t*-test.

## Supplementary information

Supplemental Table 1

Supplemental Table 2
